# Thermal Runaway Suppression Mechanism of Thermosensitive Microcapsules for Lithium-Ion Batteries

**DOI:** 10.3390/polym17172374

**Published:** 2025-08-31

**Authors:** Zujin Bai, Pei Zhang, Furu Kang, Zeyang Song, Yang Xiao

**Affiliations:** 1 School of Safety Science and Engineering, Xi’an University of Science and Technology (XUST), Xi’an 710054, China; baizj2022@xust.edu.cn (Z.B.); zhangpei@stu.xust.sdu.cn (P.Z.); xiaoyang@xust.edu.cn (Y.X.); 2 Key Laboratory of Mine Exploitation and Disaster Prevention in Western China (XUST), Ministry of Education, Xi’an 710054, China

**Keywords:** lithium-ion batteries, thermosensitive microcapsules, in situ polymerization, thermal runaway of lithium-ion batteries, synergistic effects

## Abstract

Lithium-ion batteries (LIBs) have garnered extensive application across various domains. However, frequent safety incidents associated with these LIBs have emerged as a significant impediment to their further advancement. Consequently, there is an urgent necessity to develop a novel fire extinguishing agent that possesses both rapid fire suppression and efficient cooling capabilities, thereby effectively mitigating the occurrence and propagation of fires in LIBs. This study pioneers the development of an adaptive thermosensitive microcapsule (TM) fire extinguishing agent synthesized via in situ polymerization. The TM encapsulates a ternary composite core—perfluorohexanone (C_6_F_12_O), heptafluorocyclopentane (C_5_H_3_F_7_), and 2-bromo-3,3,3-trifluoropropene (2-BTP)—within a melamine–urea–formaldehyde (MUF) resin shell. The TM was prepared via in situ polymerization, combined with FE-SEM, FTIR, TG–DSC, and laser particle size analysis to verify that the TM had a uniform particle size and complete coating structure. The results demonstrate that the TM can effectively suppress the thermal runaway (TR) of LIBs through the synergistic effects of physical cooling, chemical suppression, and gas isolation. Specifically, the peak TR temperature of a single-cell LIB is reduced by 14.0 °C, and the heating rate is decreased by 0.17 °C/s. Additionally, TM successfully blocked the propagation of TR thereby preventing its spread in the dual-LIB module test. Limitations of single-component agents are overcome by this innovative system by leveraging the ternary core’s complementary functionalities, enabling autonomous TR suppression without external systems. Furthermore, the TM design integrates precise thermal responsiveness, environmental friendliness, and cost-effectiveness, offering a transformative safety solution for next-generation LIBs.

## 1. Introduction

In the energy-intensive modern society, LIBs characterized by an environmentally friendly nature and high-voltage platforms have been extensively applied in electronic devices and electric vehicles, serving as a critical medium for energy storage [1]. However, the safety concerns surrounding LIBs have garnered increasing attention, with thermal runaway (TR) emerging as a particularly prominent issue. A series of complex exothermic reactions will be triggered when LIBs are affected by external factors such as overcharging, over-discharging, and short-circuiting with mechanical damage or by internal impurities, dendrite growth, and other defects [2,3,4]. These reactions persist, leading to rapid heat accumulation, which causes a sharp increase in LIB temperature and may ultimately trigger TR [5,6]. This can further result in LIBs’ combustion or even explosion, posing a significant threat to people’s lives and property [7]. Therefore, efficient and timely fire suppression following TR in LIBs has emerged as a critical research focus to ensure their safe application.

Managers and researchers have conducted extensive studies to address this issue in the fields of scientific research and LIBs, yielding highly significant outcomes. Traditional fire-fighting methods primarily utilize the heat-absorbing properties of water to dissipate heat and deprive the combustion process of oxygen, thereby suppressing the propagation of uncontrolled fires. Said et al. [8] investigated the suppression of TR of LIBs with different flow rates of water-mist extinguishing agent. Wang et al. [9] explored the effect of water mist containing compound additives on LIB fires. Ditch [10] investigated the effect of a water spraying system on cardboard box applications in LIB silos. Lou et al. [11] argued that foam extinguishing agents, a type of liquid extinguishing agent, react with free radicals on the surface of the cell to form stabilized substances and water vapor, thus further suppressing LIB fires but not preventing the continuation of the TR chain reaction. To summarize, the above results show that the conductivity of water can short-circuit LIB modules or electrical equipment and may cause reignition, limiting the application of water-based extinguishing agents in LIB fires. Zhang et al. [12,13,14] proved that high-boiling chemical gaseous extinguishing agents have become a hot research topic by virtue of their low critical extinguishing concentration, rapid temperature reduction, and environmental friendliness. However, the high cost associated with perfluorohexanone, which is 5 to 10 times more expensive than dry powder, along with the requirement for high-pressure storage, has restricted its large-scale application [15,16,17]. Ke et al. [18] confirmed that a halon fire extinguishing agent can be used to put out fires in electrical and electronic equipment, storage facilities, and vehicles, but it is banned because it damages the ozone layer. Zhou et al. [19] concluded that the inert gases such as CO_2_ without residual pollution require a high extinguishing concentration and insufficient cooling capacity. But it makes them hard to be applied and blocks the TR chain in the LIBs. Wang et al. [20] found that heptafluoropropane has a high latent heat of vaporization and is effective in extinguishing fires at a high cost. This substance can be used as a substitute for a halon extinguishing agent. However, its application in controlling LIB fires is limited by its finite cooling capacity and inability to prevent TR, which may lead to secondary LIB fires. Rohilla et al. [21] concluded that although aerosols can effectively suppress LIB flames in confined spaces, they exhibited limited efficacy in mitigating the propagation of TR. Huang et al. [22] concluded that liquid nitrogen was effective in suppressing TR propagation. Nonetheless, this suppression effect diminished as the surface temperature of the cell increased. Zhou et al. [23,24,25] and other experts believed that the traditional dry powder fire extinguishing agent has high extinguishing efficiency and low cost, but the cooling displayed a poor performance and the residue easily corrodes the equipment. And the ultra-fine dry powder is unable to solve the problem of TR at the deep level even though it improves the fire extinguishing efficiency by 6–10 times [26,27].

To solve the above problems of fire extinguishing agents, more experts and scholars will focus on microencapsulated fire extinguishing agents. Microcapsule fire extinguishing agents, representing a novel generation of environmentally friendly fire protection technology, have achieved significant advancements in material design encapsulation techniques and application contexts over the past few years. In the realm of technological research and development, the double-layer polymer TM has markedly improved the stability and release efficiency of the fire extinguishing agent through the optimization of core material encapsulation efficiency and thermal response performance. For instance, the gelatin–urea–formaldehyde resin bilayer microcapsules developed by Lee et al. [28] have a core loss rate of only 0.005% after 100 days of storage, and the time to extinguish the fire is 33% shorter than the single-layer structure, while core–shell synergy enhances the accuracy of the high-temperature-triggered release. In the field of encapsulation for environmentally friendly fire extinguishing agents, core materials with low global warming potential (GWP) and zero ozone depletion potential (ODP), such as perfluorohexanone, heptafluorocyclopentane, and methoxy-nonafluorobutane, have become dominant. These materials comply with EU F-gas regulations due to their relatively low GWP values, which contrasts sharply with the high GWP and ODP of previously phased-out halon gases. The microencapsulation of these materials has effectively addressed the issue of volatility. As an illustration, perfluoro (2-methyl-3-pentanone) microcapsules prepared by Gui et al. [29], who conducted in situ polymerization, showed excellent TR suppression in LIBs while maintaining a stable LIB electrochemical performance. In terms of application scenarios, microcapsule technology has been effectively incorporated into both the electrolyte and separator of LIBs, thereby improving their performance and stability. The F-500 microcellular capsule fire extinguishing agent reported by Meng et al. [30] captured electrolyte organic solvents via micellization behavior. This agent has been shown to reduce the fire extinguishing time of a 243 Ah LiFePO_4_ battery from 337 to 49 s and to decrease the heat of combustion by 10 MJ. In summary, the novel microcapsule technology has opened up new avenues for LIB safety protection [31]. A fast-responding TM fire extinguishing agent was designed based on existing microencapsulation technology. TM fire extinguishing agents consist of fire extinguishing core materials encapsulated by organic wall materials. TMs are capable of effectively containing and preserving fire extinguishing substances. Upon being formulated into TM, the fire extinguishing agent leverages advantages such as its small size, which facilitates access to the flame region, as well as the covering and flame-retardant suppression effects provided by the wall material. Consequently, the efficiency of fire extinguishing can be substantially enhanced [32,33,34].

Accordingly, an optimal LIB fire extinguishing agent must possess superior thermal dissipation properties and highly efficient flame inhibition efficacy, excellent electrical insulation characteristics and environmental benignity, and economic viability thereby ensuring comprehensive fire safety and sustainability. Fluorine-containing derivatives have gradually received widespread attention due to unique advantages in suppressing the combustion of LIBs, which can effectively cut off the combustion chain reaction without affecting the normal operation of electrical equipment. This investigation systematically examines how the use of MUF resin is characterized by high stability, excellent density, and superior mechanical strength. The shell material ensures the TM stability, responsiveness, and fire suppression performance under repeated thermal cycling and long-term storage in practical applications [35]. Perfluorohexanone and other composite materials were utilized as the core materials for the preparation of TM fire extinguishing agents. Concurrently, it was proposed that the TM fire extinguishing agent be uniformly applied to the surface of the LIBs, thereby forming a ‘protective coating’. The fire extinguishing strategy is that the TMs rupture and quickly release the composite fire extinguishing material when the LIBs experiences thermal abnormalities. And this study pioneers a pressure-free, eco-friendly TM system via in situ polymerization, encapsulating a ternary composite core—C_6_F_12_O, 2-BTP, and C_5_H_3_F_7_—within an MUF resin shell. The design achieves dual breakthroughs: (1) Microencapsulation resolves traditional agents’ volatility/corrosion while enabling on-demand release at 120 °C through intelligent thermal triggering. The 120 °C threshold embodies a meticulously calibrated trade-off between preventing premature leakage and ensuring activation reliability, directly contributing to the efficacy of TM as a self-responsive, eco-friendly LIB fire protection solution. (2) Ternary synergy overcomes single-agent limitations by integrating physical cooling, chemical suppression, and gas isolation. This integrated approach achieves a higher temperature reduction efficiency than water mist and a faster cooling speed than inert gases, enabled by autonomous 120 °C triggering.

The proposed TM fire extinguishing method effectively resolves limitations of traditional methods documented in this work, particularly their delayed response and inefficient fire suppression. TM’s autonomous activation complements BMS monitoring capabilities, forming a closed-loop safety system with faster reaction times than conventional agent injection methods. Future work will prototype this integration in multi-module battery packs.

## 2. Experiment and Methods

### 2.1. Preparation of TM

#### 2.1.1. Material Preparation

**Figure 1:**
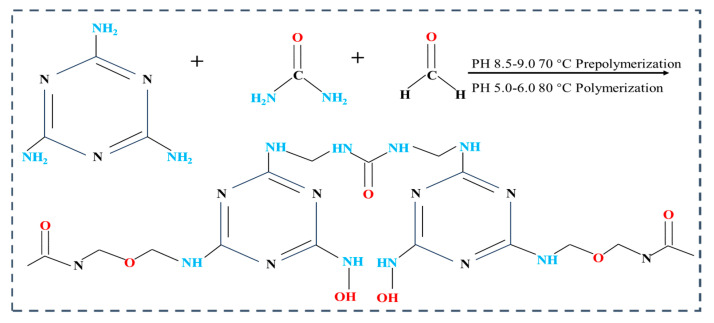
Polymerization of urea with formaldehyde.

The chemical reagents specially selected in this work were all commercially available at an analytical grade without further purification. Based on the development requirements for clean fire extinguishing agent systems, a ternary composite core material system was constructed [36]. This system utilized perfluorohexanone (C_6_F_12_O, 98%, supplied by Shanghai Haohong Biomedical Technology Co., Ltd., Shanghai, China), 2-bromo-3,3,3-trifluoropropene (2-BTP, ≥80%, provided by Shanghai Aladdin Bio-Chem Technology Co., Ltd., Shanghai, China), and heptafluorocyclopentane (C_5_H_3_F_7_, >95%, sourced from Shandong Keyuan Biochemical Co., Ltd., Laizhou, Shandong, China). During the experiment, 1-octanol (purity 99.5%, Shanghai Macklin Biochemical Technology Co., Ltd., Shanghai, China) was employed as an antifoaming agent to prevent the excessive formation of bubbles induced by mechanical stirring. Melamine (RON, 99%, Shandong Keyuan Biochemical Co., Ltd., Shandong, China), formaldehyde solution (McLean, 37% aqueous solution, Shanghai McLean Biochemical Technology Co., Ltd., Shanghai, China), and urea (RON, 99%, Shandong Keyuan Biochemical Co., Ltd., Laizhou, Shandong, China) were used to prepare prepolymer. This thermosetting material is synthesized via the polycondensation reaction of urea and formaldehyde, catalyzed by an acidic agent. The polymerization mechanism follows the reaction pathway illustrated in Figure 1 [37], and the microencapsulation of the core material is ultimately achieved through in situ polymerization.

#### 2.1.2. Sample Preparation

**Figure 2:**
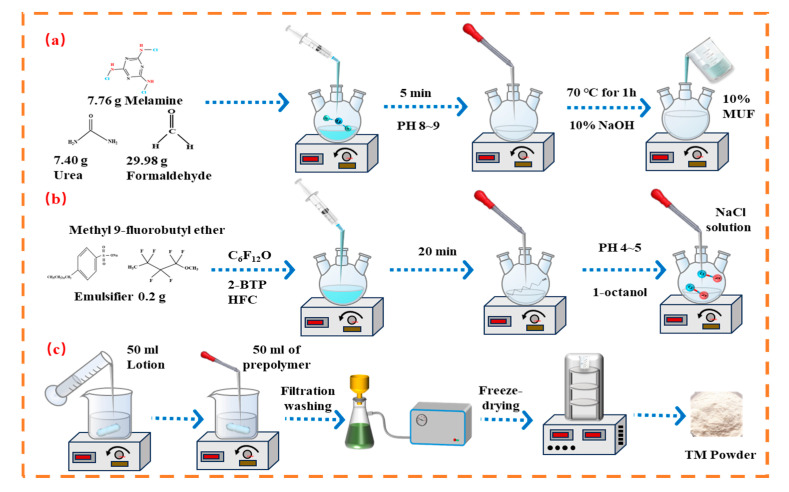
Flow chart for the preparation of C_6_F_12_O-modified TM. (**a**) Preparation of MUF; (**b**) preparation of emulsion; (**c**) preparation of TM.

Through systematic optimization, TM preparation requires three steps as follows:

Synthesis of MUF Prepolymer: In a three-neck, round-bottomed flask, 7.76 g of melamine, 7.40 g of urea, 29.98 g of formaldehyde solution, and 30 g of deionized water were sequentially added under stirring conditions and thoroughly mixed to initiate the synthesis process. The solution was stirred at 1000 rpm for 5 min to ensure the complete dissolution of the monomer. Subsequently, the pH was adjusted to 8.5–9.0 using a 10% sodium hydroxide solution. Under continuous stirring conditions, gradually increase the temperature from 30 °C to 70 °C at a rate of 2.5 °C/min in a constant-temperature water bath. After being stirred at 70 °C for 1 h, the mixture was subsequently cooled to below 40 °C in an ice-water bath. The resultant prepolymer was diluted with 375 g of distilled water to prepare a 10 wt% MUF prepolymer solution, as shown in Figure 2a.

Emulsion Preparation: A 250 mL three-necked flask containing 200 mL deionized water was charged with 5 mL C_6_F_12_O, 5 mL 2-BTP, 5 mL C_5_H_3_F_7_, 0.2 g emulsifier, and sufficient methyl perfluorobutyl ether. Initial homogenization was performed at 1000 rpm, followed by emulsification at varying speeds for 20 min. During the process, a controlled amount of 1-octanol was added dropwise to eliminate the bubbles formed as a result of vigorous stirring. At low rotational speeds, the emulsion was stabilized through the gradual addition of a 4 wt% sodium chloride solution over a 5 min period, as shown in Figure 2b.

TM Preparation: Under continuous stirring, adjust the pH of the emulsion to 4.0–5.0 using a 10% citric acid solution. Subsequently, 50 mL of a 10 wt% MUF prepolymer solution was gradually added to the emulsion under continuous stirring. The mixture reacted for 2 h at 70 °C. The resulting product was subjected to suction filtration and subsequently underwent two washing cycles, the first with petroleum ether and the second with deionized water. It was then transferred to a freeze-drier for 24 h, yielding TM powder, as shown in Figure 2c.

### 2.2. Characterization Testing

It is crucial to characterize the morphological structure and chemical properties of TM for the purpose of evaluating the particle size and chemical composition of the prepared TM. The surface morphology, molecular structure, heat storage performance, thermal stability, and particle size distribution of the TM were comprehensively characterized using Field-Emission Scanning Electron Microscopy (FE–SEM), Fourier Transform Infrared Spectroscopy (FTIR), Thermogravimetric Analysis (TGA), Differential Scanning Calorimetry (DSC), and a laser particle size analyzer. These analyses provide robust support for further optimizing their action effects.

#### 2.2.1. FE–SEM Testing

The scanning electron microscope (SEM) employed, a Zeiss Sigma model (Zeiss (Carl Zeiss AG), Oberkochen, Germany), features a platinum (Pt) gold spray target and a Schottky field emission electron gun. The SEM achieves resolutions of 0.9 nm at 15 kV and 2.0 nm at 30 kV, with magnification ranging from 8× to 100,000×. A small quantity of the sample is directly affixed to conductive adhesive and subjected to 45 s of gold spraying by using the Quorum SC7620 sputter coater (Quorum Technologies, Laughton, United Kingdom) to enhance electrical conductivity. The three-dimensional morphological characteristics of the TM surface are captured via the SE/BSE detector within the microscope column, focusing the analysis on the compactness of the shell layer, the dispersion of the spheres, and the structural integrity.

#### 2.2.2. FTIR Testing

FTIR spectroscopy analysis was performed using a WQF–530A spectrometer (Beijing Beifeng Ruili Analytical Instrument Co., Beijing, China), a versatile analytical platform capable of detecting chemical functional groups in solid, liquid, and gaseous phases. The system incorporates a deuterated L-alanine-doped triglycine sulfate detector with enhanced sensitivity. Prior to sample analysis, background spectra were systematically collected to eliminate environmental interference. Subsequently, KBr and TM samples were uniformly dispersed on the crystal surface at a mass ratio of 200:1 for subsequent testing. The wavelength range for the entire FTIR analysis spans from 4000 cm^−1^ to 500 cm^−1^.

#### 2.2.3. Thermal Property Testing

The correlation between sample mass loss and temperature variations across different reaction stages was investigated by a NETZSCH TG(NETZSCH, Selb, Germany) analyzer from Germany under a nitrogen purge gas environment at an inlet speed of 50 mL/min over a heating temperature range from 25 to 800 °C and a heating rate of 20 °C/min. Sample masses were maintained at 2.0 ± 0.1 mg, ensuring consistent relative filling amounts and particle sizes in the crucible before testing.

The thermal properties of the TM samples were evaluated by employing a DSC from the German company NETZSCH(NETZSCH, Selb, Germany), specifically the DSC 214 model. A precisely weighed quantity of 5–10 mg of fully dried TM powder was carefully placed in an alumina crucible. The instrument was operated under a nitrogen atmosphere with a heating rate of 5 °C/min. Upon initiation of the test, the instrument simultaneously recorded the mass change and thermal effect of the sample. The thermal properties of the samples were subsequently determined by comparing thermogravimetry (TG) curves and DSC curves.

#### 2.2.4. Particle Size Testing

Laser particle size analyzers are extensively utilized for the precise measurement of TM sample particle sizes. The primary function of such an instrument lies in ensuring the uniformity of the particle size distribution of TM samples. By systematically screening the samples and excluding those that deviate from the specified particle size range, the quality and performance of TM products can be effectively ensured. During the experiment, it is crucial to carefully select TM fire extinguishing agent samples that exhibit uniform dispersion characteristics. Extract a small amount of these samples and add them to the dispersant. Then, the Microtrac S3500 laser (Microtrac MRB, Largo, FL, USA) particle size analyzer was conducted to explore the particle size distribution of the prepared TM products. Ultimately, a fitting analysis was performed on the acquired particle size distribution data, thereby enabling the successful derivation of the particle size distribution curve.

### 2.3. TR Experiment of LIBs

Given the substantial explosive risk associated with LIB TR experiments, these tests must be conducted in a completely enclosed and personnel-free environment to ensure safety [38,39]. The LIBs were fixed in the explosion-proof room, and the explosion-proof door was securely closed to prevent any potential irreversible harm to experimenters and experimental equipment.

#### 2.3.1. Experimental Device for TR of LIBs

**Figure 3:**
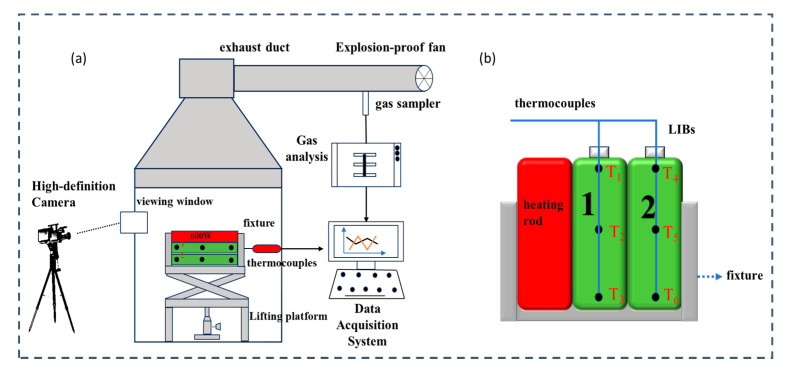
Experimental diagram of TM suppressing TR of LIBs. (**a**) Diagram of the experimental setup; (**b**) arrangement of heating rod and LIBs and distribution of thermocouples.

The experimental system setup was independently designed and is described in Figure 3a. It comprises multiple components, including an explosion-proof combustion chamber and a heating rod, thermocouples, a data acquisition system, and high-definition cameras. These components are specifically arranged to enable real-time monitoring of temperature changes, dynamic behavior of the LIBs during TR, and heat transfer between adjacent LIBs, thereby facilitating a comprehensive analysis and safety assessment of the TR process. To ensure reliability, all tests were repeated five times.

(1) A stainless steel, explosion-proof combustion chamber equipped with a smoke exhaust system and an observation window.
(2) An INR 18650 LIB, a 200 W electric heating rod (EVE Energy, Huizhou, Guangdong Province, China), and three K-type thermocouples with a diameter of 1 mm were used in the experiment. The thermocouples were positioned at the top, middle, and bottom of the upper surface of the LIBs and secured using high-temperature tape, aluminum foil tape, and wire to prevent detachment in the event of a LIB explosion. The LIB cells were labeled as *T*_1_, *T*_2_, and *T*_3_ for cell 1, and *T*_4_, *T*_5_, and *T*_6_ for cell 2. The heating rod was connected in parallel with the LIBs.
(3) Data acquisition system. This system is capable of monitoring temperature changes in real time throughout the TR process of LIBs, providing critical data for analysis and safety assessment.
(4) High-definition cameras are utilized to record the dynamic changes in LIBs during TR in real time, including temperature fluctuations, morphological transformations, and electrolyte ejection behavior.
(5) The specific arrangement of the heating rods is illustrated in Figure 3b. Prior to actual operation, the power cords should be completely wrapped with aluminum foil tape to ensure safety and mitigate potential risks associated with flames or flying sparks. Subsequently, activate the heating rod switch to initiate the heating process for LIB No. 1, thereby inducing a TR phenomenon under controlled experimental conditions. After the power supply to the heating rod was disconnected, the No. 1 LIB, which had undergone a combustion explosion, continued to release heat. This resulted in the temperature of the adjacent No. 2 LIB increasing steadily, thereby facilitating heat transfer between the two LIBs. The LIB fixation device was constructed using iron wire and cable ties.

#### 2.3.2. Experimental Steps for TR of LIBs

The experiments designed to suppress TR propagation in INR18650 LIBs were classified into two categories: single-cell LIB and dual-LIB module TR experiments. The specific procedures are outlined as follows:

(1) The TM fire extinguishing agent is prepared and formulated into adhesive fire extinguishing stickers, which can subsequently be applied to the surface of the LIBs [40]. TM achieves a uniform coating coverage on the LIB cell body surface, reaching a 95% coverage rate.
(2) The constant-temperature and -humidity chamber, in conjunction with the charge–discharge instrument, maintains the LIBs at a constant temperature of 40 °C during testing. Each test LIB is discharged at a constant current of 1 C until the voltage reaches 2.75 V and is then charged to a 100% state of charge using a constant current of 1 C. The risk of TR is most significant when the LIB is fully charged, as this condition illustrates the cooling performance of the material under extreme conditions.
(3) Secure the LIB by firmly attaching it to the heating rod using iron wire and cable ties. This arrangement ensures stability and prevents potential issues, such as explosion or detachment during operation.
(4) Turn on the equipment, including electric heating rods, data acquisition systems, high-definition cameras, and thermocouple systems, and heat the LIBs until TR occurs.
(5) Observe the experimental phenomenon and record the data. When the temperature of LIBs exceeds 120 °C, the core material of the TM fire extinguishing agent coated on the surface of the LIBs is automatically released, effectively reducing the temperature and mitigating TR risks. When the temperature decreases to within the safe threshold, the data acquisition system automatically records the temperature variations throughout the suppression process. The response time, release time, suppression duration, and extended TR suppression time of the LIBs encapsulated with TM were systematically analyzed and recorded to successfully complete the experiment.
(6) Repeat the aforementioned steps and utilize natural cooling methods to mitigate TR in LIBs.

## 3. Results and Discussion

### 3.1. Preparation Strategies and Extinguishing Methods of TM Fire Extinguishing Agent

This research pioneered the development of a TM-based fireproof system with self-responsive characteristics. This technology fabricates a MUF-coated, core–shell-structured TM via in situ polymerization. Its core is composed of a ternary composite fire extinguishing agent consisting of C_6_F_12_O, 2-BTP, and C_5_H_3_F_7_. When the LIBs undergo TR, the resulting temperature increase causes the TM wall material to rupture, thereby enabling the self-driven release of the fire extinguishing agent. Its mechanism of action is defined by dual response properties. (1) Fluoroalkane compounds suppress chain combustion reactions by means of chemical quenching. (2) The highly endothermic process effectively mitigates the surface temperature rise of the LIBs, thereby enhancing thermal stability. This technology overcomes the limitations of conventional safety measures, while preserving the electrochemical performance of the LIBs, and it establishes a non-intrusive intelligent fire prevention system. It provides an innovative solution for mitigating the spread of fires in high-energy-density LIB systems.

### 3.2. Characterization of Microscopic Properties

#### 3.2.1. Microcosmic Properties of TM Fire Extinguishing Agents

**Figure 4:**
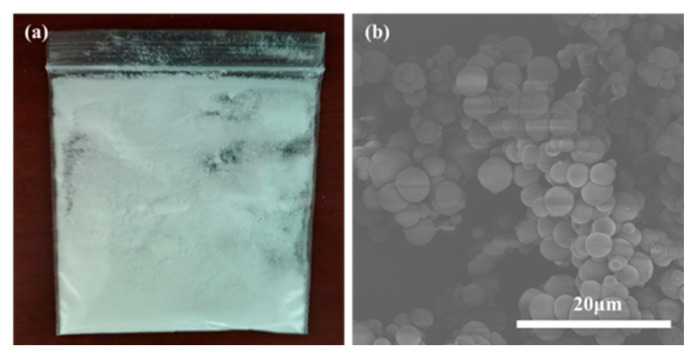
TM sample topography. (**a**) Physical image of TM; (**b**) FE-SEM of TM (magnification: 5000×).

FE-SEM characterization was conducted to analyze the detailed particle size as well as the chemical structure of the TM. The morphology and quality of the TM were observed by adjusting the ratio of the core to the wall material to determine the optimal encapsulation conditions during the preparation of the TM [41]. As shown in Figure 4a, the TM was prepared under optimal conditions and exhibited a white powder form. This characteristic is attributed to the water solubility of the wall material MUF, which facilitates the emulsification process by enabling oil-phase droplets to be readily dispersed into smaller droplets, thereby leading to reduced TM particle sizes. The MUF wall material was formed by the three monomers of melamine, urea, and formaldehyde through the formation of the MUF prepolymer by prepolymerization under the alkaline condition, and then cross-linking and a condensation reaction took place in the acidic condition. This structure enhances the mechanical strength of the MUF thermally conductive TM, reducing the likelihood of forming TMs with relatively larger particle sizes. Figure 4b presents the results of field emission SEM analysis. FE-SEM showed a regular spherical morphology. The dense shell effectively ensures that the core material remains encapsulated without leakage. The dispersion is also relatively favorable, suggesting that MUF has formed an effective coating on the composite core material. It provides a certain degree of protection for the core material, suppresses volatilization, and reduces the likelihood of toxic gas generation.

#### 3.2.2. FTIR Spectrum of TM

**Figure 5:**
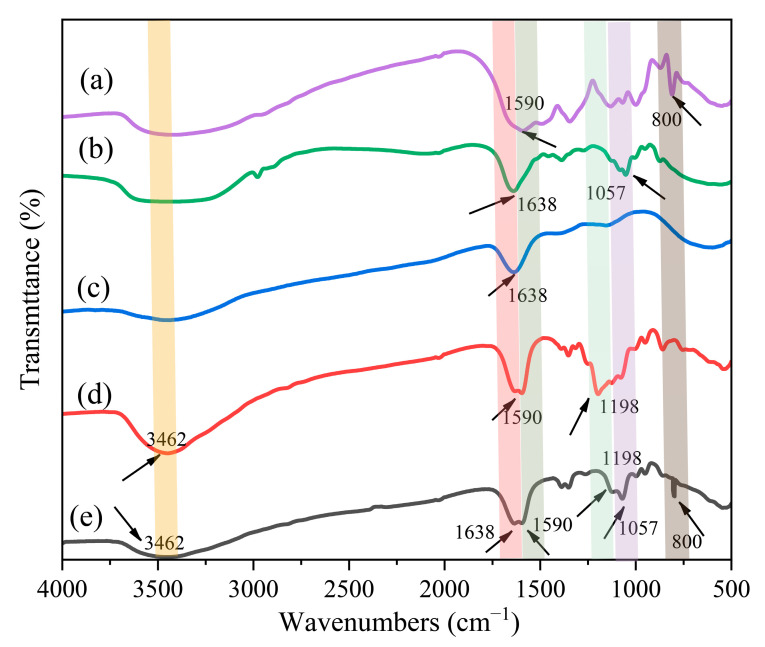
FTIR analysis of individual components in TM formulations. (**a**) FTIR of C_6_F_12_O; (**b**) 2-BTP; (**c**) C_5_H_3_F_7_; (**d**) MUF; (**e**) TM extinguishing agent.

From the FTIR characteristic peak in Figure 5a, it is evident that the absorption peak at 1590 cm^−1^ corresponds to the C=O stretching vibration of C_6_F_12_O, while the absorption peak at 800 cm^−1^ is attributed to the CH bending vibration [42]. As shown in Figure 5b, the prominent and intense peak at 1638 cm^−1^ is attributed to the C=C stretching vibration absorption peak characteristic of alkanes, while the peak at 1057 cm^−1^ corresponds to the C–O stretching vibration absorption peak. In Figure 5c, only one peak is observed, corresponding to the C=C stretching vibration at 1638 cm^−1^. Three distinct peaks can be observed in Figure 5d. The absorption peak at 3462 cm^−1^ is assigned to the stretching vibration of the hydroxyl (OH) group in alcohols. The peak at 1590 cm^−1^ corresponds to the stretching vibration of the carbonyl (C=O) bond, while the peak at 1198 cm^−1^ is attributed to the combined bending and stretching vibrations of the carbon–carbon (C–C) bond. The location and intensity of the absorption peaks of the TM are similar to the superposition of the core material and the wall material from Figure 5e. The absorption peaks of the core material are at 1590 cm^−1^, 1638 cm^−1^, 1050 cm^−1^, and 800 cm^−1^. And absorption peaks of the wall material MUF appeared at 3462 cm^−1^, 1590 cm^−1^, and 1198 cm^−1^. This suggests that C_6_F_12_O, 2-BTP, C_5_H_3_F_7_, and MUF are likely components of the TM. There are few new infrared absorption peaks in Figure 5e, which suggests that the three complex cores were successfully encapsulated by the MUF by in situ polymerization [43].

#### 3.2.3. Encapsulation Properties of TM

**Figure 6:**
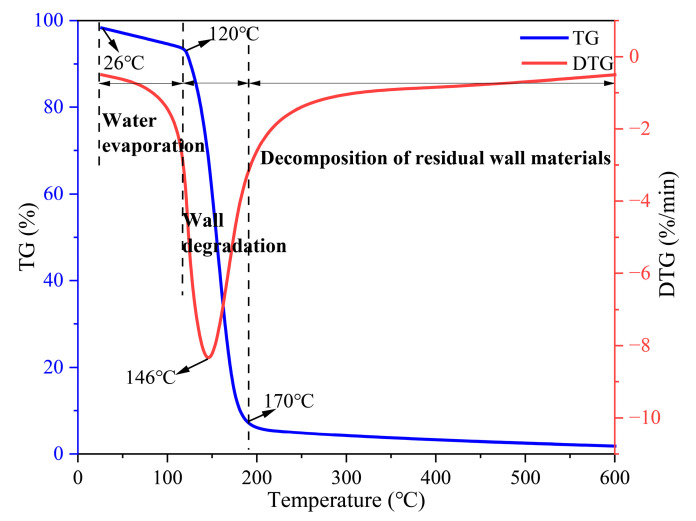
TG-DTG plot of wall MUF and TM extinguishing agent.

**Figure 7:**
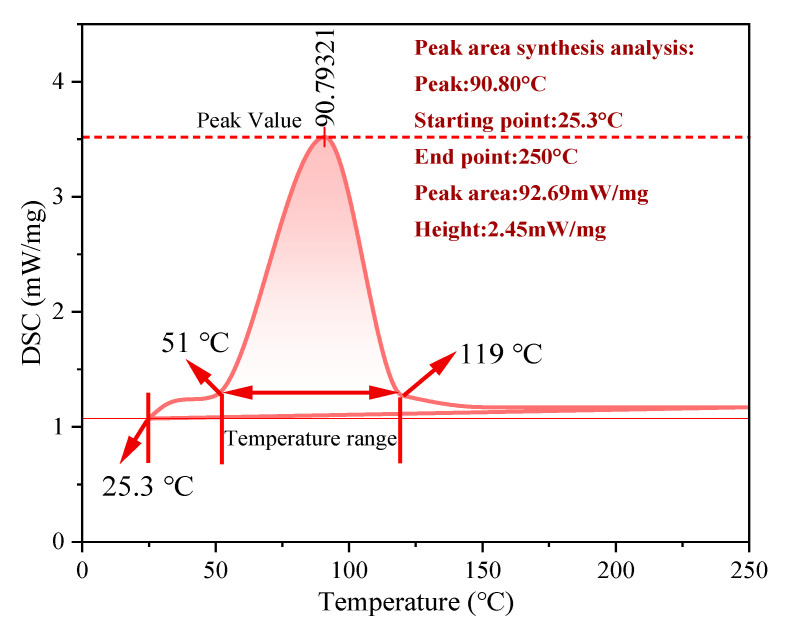
DSC curves of TM.

The TG–DTG thermograms are depicted in Figure 6, which indicates that the thermal decomposition process of the TM fire extinguishing agent occurs in three distinct stages [44]. The initial phase is characterized by water evaporation, during which the wall material and TM undergo a mass reduction of approximately 5%. In the second stage, the wall material undergoes MUF decomposition, concurrent with the release of the core material. During this process, the wall material experiences an 85% mass loss, while the TMs lose up to 90% of mass due to the evaporation of the core material. The peak of maximum mass loss in the DTG curve of the TM suggests that the dense shell material effectively retards the decomposition of the core material. In the third stage, the residue decomposed slowly with a mass loss of only 5%, indicating that MUF effectively encapsulated the core material and confirming the stability of the high-temperature residue of the material. The DSC curve presented in Figure 7 further validates the effectiveness of the coating [45]. The TM displays a broad endothermic peak ranging from 51 to 119.0 °C, with a maximum peak at 90.8 °C. This endothermic behavior is primarily attributed to water evaporation, minor leakage of the core material, and resin degradation. Upon exceeding a temperature of 119 °C, the structural integrity of the wall material is compromised, resulting in the subsequent release of the core material. The endothermic temperature range spans 68 °C, suggesting that the TM can be stably stored at temperatures below 120 °C. Simultaneously, the cooling effect is realized through continuous heat absorption via the phase transition and decomposition of the core material, making it suitable for scenarios requiring delayed release of fire extinguishing agents. The TM system demonstrates multifunctional efficacy through its engineered architecture. It integrates core-material protection with on-demand release capabilities while maintaining exceptional thermal stability coupled with active cooling functionality. The encapsulating wall structure ensures optimal core-component integrity under ambient conditions and enables precise thermally induced activation of TM fire extinguishing agents during pyrolysis events.

#### 3.2.4. TM Particle Size Analysis

**Figure 8:**
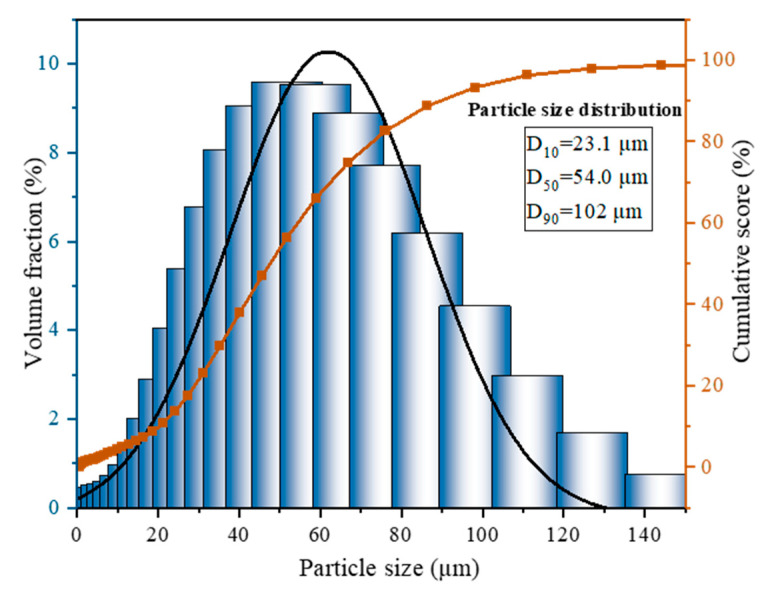
Particle size distribution of TM.

The particle size of TM is not only closely related to the fire extinguishing effect but also affects the storage stability and lifespan of the product. Figure 8 shows the particle size distribution of the TM fire extinguishing agent prepared. D_10_, D_50_, and D_90_ are, respectively, 23.1 μm, 54.0 μm, and 102 μm for TM.

The particle size distribution (Figure 8) showed D_10_ = 23.1 ± 1.2 μm, D_50_ = 54.0 ± 2.5 μm, and D_90_ = 102 ± 4.8 μm (uncertainty: 5% confidence interval, *n* = 5). This normal distribution confirms homogeneity, with minor aggregation attributed to humidity during testing. This results indicate that the particle sizes of the TM were mainly distributed between 2 and 185 μm, and the majority of particles were mainly clustered around 53 μm. Particles were homogeneous, with an average size of 54 μm (specific surface area: 223.6 m^2^/kg), exhibiting a normal distribution. However, a small number of particles exhibited larger sizes due to water absorption during experiments, leading to adhesion and aggregation. The measured TM particle size results are roughly consistent with the particle size results obtained from SEM images, indicating that the TMs synthesized by in situ polymerization have an outstanding surface morphology and a relatively uniform particle size distribution.

### 3.3. Characteristics of TM in Suppressing TR of LIBs

**Figure 9:**
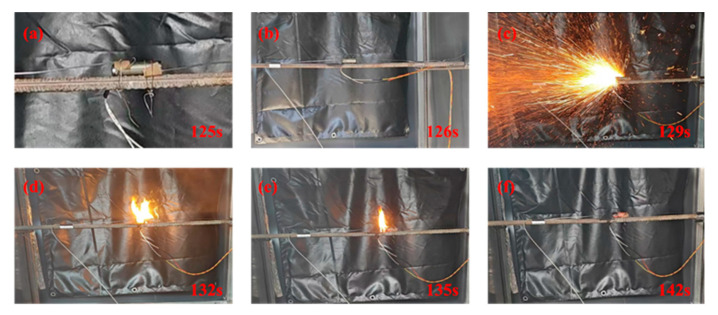
Experimental process of TR of LIBs. (**a**) Initial temperature rise with internal side reactions; (**b**) safety valve rupture with massive smoke ejection; (**c**); flame forms a jet; (**d**) sustained fierce combustion; (**e**) flame recession with diminishing intensity; (**f**) final stage with cell rupture and extinguished fire.

When observing the whole experimental process, it was found that the process of TR triggered by heating of INR18650 LIBs can be divided into four key stages [46,47,48]: (a) pre-heating stage; (b) pressure relief stage; (c) TR stage; and (d) quenching recession stage, as shown in Figure 9.

Stage a: As the temperature of the LIBs rises, a series of chain side reactions are triggered within it, which gradually generate heat, electrolyte vapors, and combustible gases [49]. This in turn causes a progressive increase in the temperature and pressure inside the LIBs, as shown in Figure 9a.

Stage b: Upon sustained heating, the trigger relief valve actuates when the internal pressure within the LIBs reaches the threshold of the safety valve, thereby signifying the onset. At this point, a jet of white smoke entrapped with electrolytes is formed above the relief valve, as shown in Figure 9b. However, the discharged smoke is not very clear due to light reflection.

Stage c: TR is triggered and a large amount of flammable gas is released above the pressure relief valve, which ruptures the safety valve at its positive pole with a crunch.

**Figure 10:**
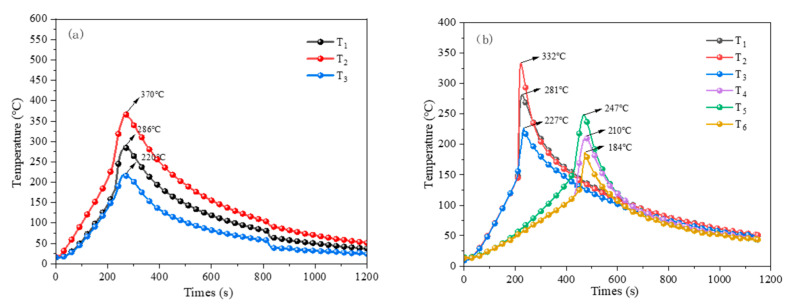
LIB TR data plot. (**a**) TR of the single-cell LIB; (**b**) TR of the dual-LIB module.

Stage d: When the temperature of the LIBs continues to rise, the positive pole releases a large number of combustible gases and electrolytes. These are ignited by the high temperature, causing an instantaneous violent explosion with dazzling white light and sparks flying in all directions. The flame forms a jet and continues to burn fiercely, as illustrated in Figure 9c–e. Figure 9f shows the end of the TR, with the LIB cell body open and the fire extinguished. The outer surface of the cell body is reddened due to high-temperature burning, and the thermocouple data acquisition is presented in Figure 10.

#### 3.3.1. Performance of TM in Suppressing TR of Single-Cell LIB

**Figure 11:**
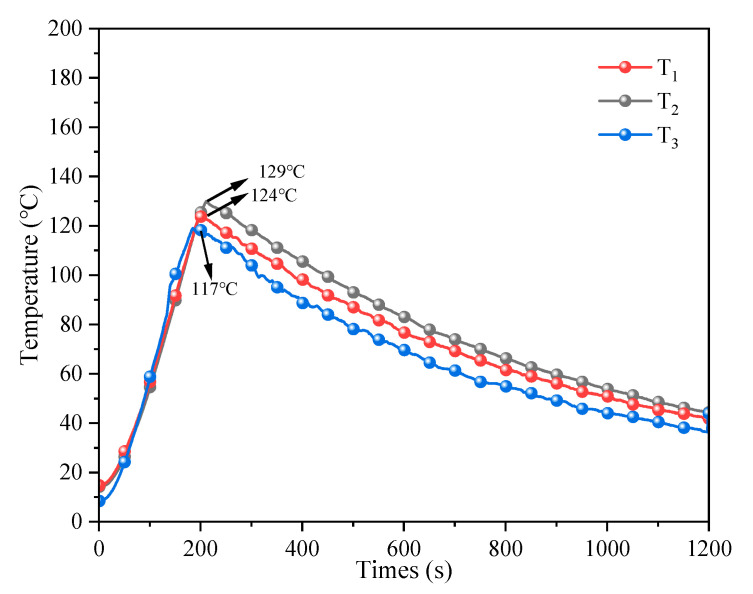
Temperature change curve of TM to suppress the early TR of the single-cell LIB.

Figure 11 displays the temperature curves of TM fire extinguishing agent to suppress the TR of a single-cell LIB. When the LIB temperature reached 120 °C, the heating rod was deactivated. It was observed that despite the application of the TM fire extinguishing agent on the surface of the LIB, an internal reaction still occurred, which led to a marginal increase in temperature. The temperature on the surface of the LIB increased from 14.2 °C to 129 °C in 210 s at an average heating rate of 0.55 °C/s. Its maximum temperature was reduced by 14 °C and the heating rate was reduced by 0.17 °C/s compared with the unused extinguishing agent, which effectively slowed the TR cycle. After the heating rod switch is turned off, the surface temperature of the cell body increases by 9 °C. The internal reaction proceeds slowly because the wall of the TM can absorb part of the heat before it breaks. As the temperature increases, the wall ruptures, releasing the wrapped extinguishing agent, which cools the cell body.

The temperature variations at the three locations within the cell body (designated as *T*_1_, *T*_2_, and *T*_3_) are relatively minor, but still the middle position has a high temperature and a more violent reaction, *T*_2_ (129 °C) > *T*_1_ (124 °C) > *T*_3_ (117 °C). Because the chemical activity of the negative electrode material is usually higher than that of the positive electrode material during the heating process of LIB, the negative electrode preferentially undergoes the oxidation reaction of de-embedded Li+ and reacts with the solid electrolyte interphase membrane. This means the negative electrode has a slightly higher temperature than the positive electrode [50]. Therefore, this TM extinguishing agent can effectively suppress the early TR of LIBs to a certain extent.

#### 3.3.2. Performance of TM in Suppressing TR of the Dual-LIB Module

**Figure 12:**
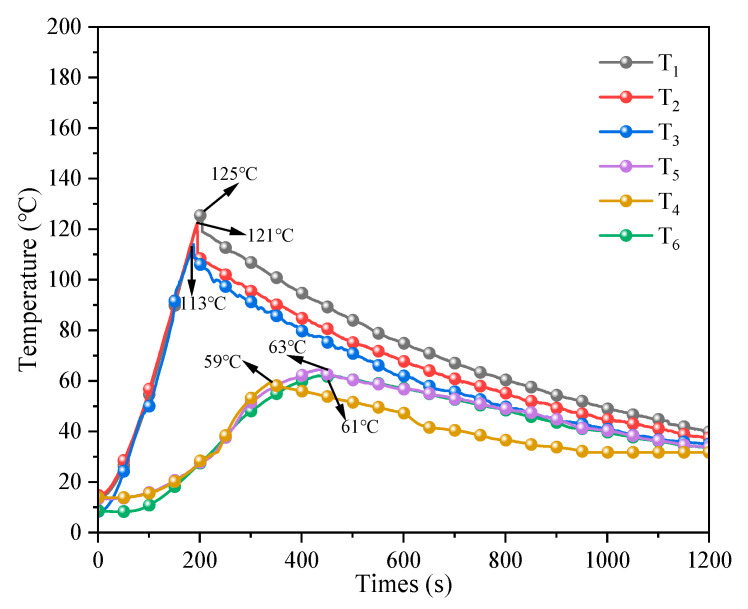
Temperature change curve of TM to suppress early TR of the dual-LIB module.

Figure 12 illustrates the temperature change in the TM suppressing the early TR of the dual-LIB module. Since the TM extinguishing agent is to suppress the early TR of the LIBs, the trigger temperature of the wall material is around 120 °C. This stops heating when the temperature of the heating rod reaches 120 °C. Although the heating was stopped, the heat was still output, and the temperature of cell body 1 slowly increased to 125.0 °C. The temperature of the middle position *T*_2_ of cell body 1 rises from 14.2 °C to 125.0 °C in 201 s, with a heating rate of 0.55 °C/s. This temperature is 18.0 °C lower than the highest temperature of natural cooling, indicating that the TM effectively slows the reaction inside the cell body and has a significant cooling effect on the suppression of the TR of the LIBs. Subsequently, cell body 1 was reduced from 125 °C to below 100 °C in 152 s, with a cooling rate of 0.16 °C/s, which was reduced by 28 s compared to the cooling time without suppression. The temperatures of the three positions of cell body 1 (*T*_1_, *T*_2_, and *T*_3_) were slightly different, *T*_2_ (125 °C) > *T*_1_ (121 °C) > *T*_3_ (113 °C). The LIB heating process triggers a cascade of internal reactions that drive TR progression. Non-uniform temperature distributions reflect material-specific reactivity: graphite anode oxidation initiates first at *T*_1_ due to SEI breakdown, while central regions (*T*_2_) accumulate heat from simultaneous electrolyte decomposition and oxygen recombination. This culminates in the TR stage, where cathode decomposition creates self-sustaining exothermic reaction loops. The heat in the middle is more concentrated under the action of thermal radiation, resulting in a higher temperature at the position of *T*_2_. In additional, *T*_1_ is the negative pole of the LIBs, which is the first to react, before the positive pole, resulting in *T*_1_ > *T*_3_. The warming of cell body 2 originates from the thermal radiation of cell body 1, and it takes 445 s to heat from 13.4 °C to 63 °C, with an average warming rate of 0.11 °C/s. Compared with the natural cooling to suppress the TR of the LIBs, the peak value is reduced by 6.0 °C, which effectively suppress the TR of the LIBs, and the temperature of cell body 2 is relatively low in general, *T*_5_ (63 °C) > *T*_4_ (61 °C) > *T*_6_ (59 °C), which is in a relatively safe state.

In summary, the TM show significant advantages in suppressing the early TR of LIBs. The TM can effectively slow the reaction rate inside cell body 1, reduce the temperature peak, and shorten the cooling time. More importantly, it also successfully prevents the spread of heat to cell body 2, minimizing the damage of TR to the entire dual-LIB module, thereby significantly reducing heat loss.

#### 3.3.3. Mechanism Analysis of TM Suppressing TR of LIBs

**Figure 13:**
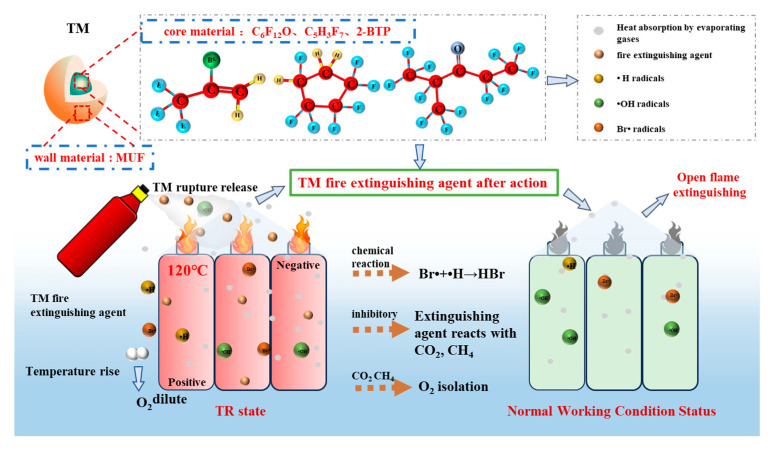
Suppression mechanism of TM to suppress TR of LIBs.

When the LIB temperature reaches 120.0 °C, the wall material MUF ruptures, causing the core materials C_6_F_12_O, 2-BTP, and C_5_H_3_F_7_ to spray out accordingly. Among these methods, chemical suppression plays a crucial role. Compounds such as C_6_F_12_O and C_5_H_3_F_7_ exhibited relatively high latent heats of vaporization. Upon release, they rapidly absorb heat and undergo phase transition to vapor, thereby effectively removing a significant amount of thermal energy. This process efficiently reduces the temperature within the LIB and its surrounding environment, thereby alleviating heat accumulation. As the temperature increases, fire extinguishing agents will generate inert gases. These gases can form a protective layer on the surface of the LIBs, effectively reducing heat transfer to adjacent areas and thus slowing thermal propagation. In addition, C_6_F_12_O and C_5_H_3_F_7_ can effectively suppress the high-temperature decomposition of the electrolyte through chemical reactions, thereby significantly reducing the formation of flammable gases. Free radicals within the combustion chain reaction can be effectively captured by the Br element in 2-BTP, thereby disrupting the oxidation process and ultimately reducing the formation of flammable gases [51]. In regard to halons or inert gases, 2-BTP remains stable below 120 °C, preventing premature decomposition during normal operation. Concurrently, the physical suppression mechanism exerts a substantial influence on this process. The interior of the LIB is filled with inert gas, which effectively reduces the concentration of O_2_. This prevents the continuation of thermal reactions, halts any further increase in temperature, and ultimately avoids the potential occurrence of fires and explosions. The synergistic action of the three-component core material of C_6_F_12_O, C_5_H_3_F_7_, and 2-BTP achieves the triple effect of physical cooling, chemical suppression, and gas isolation. The wall material maintains stability during the normal operation of the LIB and exhibits precise responsiveness exclusively at the onset of TR, thereby ensuring the appropriate release of the TM. This suppression mechanism is summarized in Figure 13.

In summary, TM effectively suppresses the early TR of LIBs through temperature sensitivity, triggering a heat-absorbing cooling chemical, physical isolation, and mechanisms that slow the propagation of TR [52]. This fire extinguishing method not only provides a rapid response to TR events but also effectively mitigates the damage inflicted on dual-LIB modules, thereby significantly enhancing the safety of LIBs.

## 4. Conclusions

This study demonstrates that TM synthesized via in situ polymerization to encapsulate C_6_F_12_O, C_5_H_3_F_7_, and 2-BTP within a MUF shell exhibited a uniform particle size distribution and precise thermal responsiveness. Systematic characterization through FE-SEM, FTIR, TG-DSC, and particle size analysis, combined with LIB TR suppression experiments, of fire extinguishing efficiency elucidates its underlying mechanism of TM. TM suppresses LIB TR through synergistic physical cooling, chemical inhibition, and gas isolation, reducing the peak TR temperature by 14.0 °C and blocking propagation in multi-cell modules. This self-responsive, eco-friendly technology offers a cost-effective solution for next-generation LIB safety.

## Data Availability

Data will be made available upon request.

## Abbreviations

The following abbreviations are used in this manuscript:

LIBs	Lithium-ion batteries
TM	Thermosensitive microcapsules
MUF	Melamine–urea–formaldehyde
C_6_F_12_O	Perfluorohexanone
C_5_H_3_F_7_	Heptafluorocyclopentane
2-BTP	2-bromo-3,3,3-trifluoropropene
TR	Thermal runaway
FE–SEM	Field-Emission Scanning Electron Microscopy
FTIR	Fourier Transform Infrared Spectroscopy
TGA	Thermogravimetric Analysis
DSC	Differential Scanning Calorimetry
SEM	Scanning electron microscope
TG	Thermogravimetry
